# Effects of Land-Use Conversion from Double Rice Cropping to Vegetables on Methane and Nitrous Oxide Fluxes in Southern China

**DOI:** 10.1371/journal.pone.0155926

**Published:** 2016-05-19

**Authors:** Ye Yuan, Xiaoqin Dai, Huimin Wang, Ming Xu, Xiaoli Fu, Fengting Yang

**Affiliations:** 1 Qianyanzhou Ecological Research Station, Key Laboratory of Ecosystem Network Observation and Modeling, Institute of Geographic Sciences and Natural Resources Research, Chinese Academy of Sciences, Beijing, China; 2 College of Life Sciences, Anhui Normal University, Wuhu, China; 3 Jiangxi Provincial Key Laboratory of Ecosystem Processes and Information, Taihe, China; 4 Department of Ecology, Evolution and Natural Resources, Rutgers University, New Brunswick, NJ 08901, United States of America; Agricultural Research Service, UNITED STATES

## Abstract

Compared with CO_2_, methane (CH_4_) and nitrous oxide (N_2_O) are potent greenhouse gases in terms of their global warming potentials. Previous studies have indicated that land-use conversion has a significant impact on greenhouse gas emissions. However, little is known regarding the impact of converting rice (*Oryza sativa* L.) to vegetable fields, an increasing trend in land-use change in southern China, on CH_4_ and N_2_O fluxes. The effects of converting double rice cropping to vegetables on CH_4_ and N_2_O fluxes were examined using a static chamber method in southern China from July 2012 to July 2013. The results indicate that CH_4_ fluxes could reach 31.6 mg C m^−2^ h^−1^ under rice before land conversion. The cumulative CH_4_ emissions for fertilized and unfertilized rice were 348.9 and 321.0 kg C ha^−1^ yr^−1^, respectively. After the land conversion, the cumulative CH_4_ emissions were −0.4 and 1.4 kg C ha^−1^ yr^−1^ for the fertilized and unfertilized vegetable fields, respectively. Similarly, the cumulative N_2_O fluxes under rice were 1.27 and 0.56 kg N ha^−1^ yr^−1^ for the fertilized and unfertilized treatments before the land conversion and 19.2 and 8.5 kg N ha^−1^ yr^−1^, respectively, after the land conversion. By combining the global warming potentials (GWPs) of both gases, the overall land-use conversion effect was minor (*P* = 0.36) with fertilization, but the conversion reduced GWP by 63% when rice and vegetables were not fertilized. Increase in CH_4_ emissions increased GWP under rice compared with vegetables with non-fertilization, but increased N_2_O emissions compensated for similar GWPs with fertilization under rice and vegetables.

## Introduction

Methane and nitrous oxide are two important greenhouse gases that have 25 and 298 times higher global warming potentials (GWPs), respectively, than CO_2_ in a time horizon of 100 years [1]. Since 1750, the atmospheric CH_4_ concentration has increased by 150% [1]. In addition, the concentration of atmospheric N_2_O has increased steadily at a rate of 0.73 ± 0.03 μg kg^-1^ yr^-1^ over the last three decades [1]. Agricultural practice is one of the major sources for these emissions, accounting for 50 and 60% of the total global CH_4_ and N_2_O emissions, respectively [2]. Agricultural practices contribute approximately 50 and 92% of the total CH_4_ and N_2_O emissions, respectively, in China [3].

Rice and vegetables are major crops grown in southern China. Because of the different growing conditions (standing water in rice vs. aerobic condition in vegetables), greenhouse gas (GHG) emissions can be different between the two cropping systems [4–6]. In standing water or anaerobic condition, CH_4_ is emitted under rice primarily through aerenchyma, diffusion, and ebullition [7]. Nitrous oxide is emitted through denitrification in water-logged condition under rice [8] and the emissions can be pronounced when flooded fields are drained or N fertilized [9]. Studies indicate that CH_4_ emissions can be higher and N_2_O emissions lower under rice than other crops [5, 10]. Nishimura et al. [4, 5] found that the cumulative CH_4_ fluxes were 2 to 14 g C m^−2^ yr^−1^ under lowland rice and ranged from −0.02 to −0.07 g C m^−2^ yr^−1^ under upland rice and the double cropping of soybean and wheat. They found that cumulative N_2_O emissions increased 4.0 to 5.3 times by changing the land-use from lowland rice to upland crops [4]. The total GWP of CH_4_ and N_2_O emissions can serve as a useful indicator for comparing the GHG impacts before and after land-use conversion [11].

Land-use conversion from rice to other crops includes variations in crop types, management practices, and changes in soil physical, chemical, and microbial properties. Straková et al. [12] demonstrated that changes in vegetation during land-use change were an important factor affecting the carbon cycle in a peatland. Crop species were also found to have a significant influence on CH_4_ and N_2_O emissions [13]. Changes in management practices can also have significant effects on GHG emissions [14–16]. For example, tillage can increase soil respiration, N_2_O emissions, and CH_4_ uptake compared with no tillage [15]. Shimizu et al. [16] found that the application of N fertilizer significantly increased N_2_O emissions compared with no application, although it had no effect on CH_4_ emissions. Changes in soil physical, chemical, and microbial properties such as soil temperature, moisture, pH, redox potential, and microbial communities can also alter CH_4_ and N_2_O emissions [17].

China has the world's second largest rice-growing area and the annual total CH_4_ emissions from Chinese rice cultivation is approximately 7.4 Tg CH_4_ yr^−1^ which is approximately 29% of the global CH_4_ emissions [18]. Large areas of rice fields have been converted to vegetable production due to the acceleration of urbanization and economic development in recent decades. China's total area under vegetable cultivation has grown from 3.5 million ha in 1980 to 17.9 million ha in 2010, whereas the area under rice decreased from 33.3 to 26.5 million ha during the same period [19]. Sun et al. [19] found that converted vegetable fields had greater soil organic C, total N, total P, and available K, but lower microbial biomass C and N than unconverted rice fields. A study conducted in the Yangtze River Delta region found that soil P fractions, especially inorganic P, increased significantly after land-use conversion from rice to other crops [20]. Such changes in soil properties during land conversion may affect CH_4_ and N_2_O emissions, but little information is available on the emission patterns and their total GWP impact, especially in southern China.

We hypothesized that land-use conversion from rice to vegetable cultivation would significantly decrease CH_4_ fluxes and increase N_2_O fluxes, but have little impact on the total GWP. The objectives of this study were to: (1) determine changes in CH_4_ and N_2_O fluxes during the conversion of land from long-term rice production to vegetables during three growing seasons from 2012 to 2013 in southern China and (2) measure the overall GWP impact of CH_4_ and N_2_O emissions. The results of this study are intended to support the China national GHG inventory for land conversion from rice to vegetable production.

## Materials and Method

### Site description

The study site was maintained by the Institute of Geographic Sciences and Natural Resources Research, Chinese Academy of Sciences. All necessary permits were obtained for this field study. The field study did not involve endangered or protected species. The experimental site was located at the Qianyanzhou Ecological Research Station (QYZ, 26°44′46″ N, 115°04′05″ E) in Jiangxi Province, southern China. The site consisted of a typical red soil (equivalent to Plinthudults in the US Soil Taxonomy) hilly region with a subtropical monsoon climate. According to the meteorological data from 1989 to 2010, the mean air temperature near the site was 18.0°C, and the coldest and warmest months were January (with the temperature range of −0.8 to 18.9°C) and July (with a temperature range of 25.1 to 30.9°C), respectively. The region receives an average total annual precipitation of 1509 mm. Double cropping of rice in a year is the primary cropping system in this area, but a large area under rice was converted to vegetable cultivation recently for economic reasons. The soil had 580, 310, and 110 g kg^−1^ sand, silt, and clay, respectively. Other soil properties at the initiation of the experiment are shown in Table 1.

**Table 1:** Soil physical and chemical properties (0−10 cm) of the study site prior to the land conversion.

Treatment^†^	Bulk density	pH	Soil organic carbon	Total nitrogen	Available P	Available K
	(g cm^−3^)		(SOC, mg g^−1^)	(TN, mg g^−1^)	(mg kg^−1^)	(mg kg^−1^)
RF	1.35±0.09^‡^	4.92±0.09	9.23±0.31	1.01±0.02	52.67±4.67	41.96±6.42
VF	1.28±0.04	5.09±0.07	9.49±0.11	1.00±0.02	48.97±2.92	40.58±3.39
RNF	1.28±0.01	5.03±0.07	9.42±0.39	1.00±0.03	48.14±3.62	56.47±8.67
VNF	1.27±0.04	4.91±0.03	9.47±0.39	0.99±0.04	51.42±4.10	54.01±10.59

^†^ Treatments are RF, rice with fertilization; NRF, rice with no fertilization; VF, vegetables with fertilization, and VNF, vegetables with no fertilization.

^‡^ Data shown are means ±standard errors for four spatial replicates.

### Experimental design

The experimental site had been continuously cultivated with rice for approximately 10 yr before land-use conversion occurred in 2012. In July 2012, we converted a portion of the area under rice to vegetable production by draining water from the field while leaving the remaining land under rice. The double-rice system, which is comprised of early rice (transplanted in April and harvested in late July) and late rice (transplanted in late July and harvested in November), was adopted as usual with a fallow in the winter. The late and early rice were transplanted at a plant population of 235000 ha^−1^ with a spacing of 25 × 17 cm. The converted vegetable areas were planted with cowpea (*Vigna unguiculata* L.) (planted in July and harvested in October) corresponding to late rice, white radish (*Raphanus sativus* L.) (planted in October and harvested in March) corresponding to fallow, and pepper (*Capsicum annuum* L.) (planted in April and harvested in July) corresponding to early rice at a population of 143000 plants ha^−1^ with a spacing of 35 × 20 cm. Each cropping system had two fertilization levels, e.g., conventional fertilization and no fertilization. Thus, the experiment included four treatments in a split plot arrangement in randomized complete block design with four replications. Land use (or crop type) was the main plot and fertilization was the split-plot treatment.

The treatments included vegetable with (VF) and without fertilization (VNF) and rice with (RF) and without fertilization (RNF). Each plot had an area of 120 m^2^ (10 m × 12 m). The rates of N, P, and K applied through urea (46% N) and compound fertilizer (15% N, 6.5% P, and 12.5% K) to early and late rice and vegetables are shown in Table 2. Fertilizer was broadcast before tillage, irrigation or rainfall. Conventional tillage was carried out at the beginning of each growing season. The vegetable plots were plowed manually with hoes and the rice plots were tilled with a plow attached to hand tractor to a depth of 20 cm. Herbicide (1.5 kg ha^−1^ metolachlor, C_15_H_22_ClNO_2_) was applied to vegetables after sowing or transplanting in each growing season. For rice, bensulfuron methyl (C_16_H_18_N_4_O_7_S, 0.007 kg ha^−1^) and butachlor (C_17_H_26_ClNO_2_, 0.26 kg ha^−1^) were applied in the third day after transplanting in each growing season. Pesticide was used on August 19, 2012, for cowpea (1.38 kg ha^−1^ Dichlorvos, C_4_H_7_Cl_2_O_4_P) and August 26, 2012, for late rice (0.31 kg ha^−1^ tricyclazole, C_9_H_7_N_3_S), while no pesticide was used for white radish, pepper or early rice. A total of 297 and 120 mm of irrigation water were applied to the rice and vegetables, respectively, during the experiment (Table 2).

**Table 2:** Management practices used for rice and vegetables.

Land-use type	Crop	Growth period	Transplanting date	Time of fertilization	N applied (kg N ha^−1^)	P applied (kg P ha^−1^)	K applied (kg K ha^−1^)	Tillage date	Tillage depth (cm)	Irrigation date	Irrigation amount (mm)
Rice	Late rice	2012−7−4~2012−11−14(Season 1)	2012−7−30	2012−7−30	71.7 (CF^†^)	31.3	59.5	2012−7−30	20	2012−7−30	60
				2012−8−10	107.5 (U^‡^)					2012−8−1	23
										2012−8−9	25
										2012−8−25	40
										2012−10−15	20
	Early rice	2013−3−28~2013−7−23(Season 3)	2013−4−24	2013−4−24	71.7 (CF)	31.3	59.5	2013−4−24	20	2013−4−24	47
				2013−5−3	107.5 (U)					2013−4−28	22
										2013−5−3	40
										2013−6−19	20
Vegetables	Cowpea	2012−7−30~2012−10−26(Season 1)	—	2012−7−30	71.7 (CF)	31.3	59.5	2012−7−30	20	2012−8−5	10
				2012−8−25	53.3 (U)					2012−8−9	15
										2012−8−12	15
	White radish	2012−10−31~2013−3−9(Season 2)	—	2012−10−30	71.7 (CF)	31.3	59.5	2012−10−31	20	2012−11−3	10
										2012−11−5	15
										2012−11−8	10
	Pepper	2013−4−7~2013−7−23(Season 3)	—	2013−4−7	71.7 (CF)	31.3	59.5	2013−4−7	20	2013−4−13	15
				2013−5−7	45 (CF)	19.6	37.3			2013−4−16	15
				2013−6−21	45 (CF)	19.6	37.3			2013−5−14	15

^†^ Compound fertilizer (Urea-based N: P_2_O_5_: K_2_O = 15%:15%:15%).

^‡^ Urea

### Measurement of the CH_4_ and N_2_O fluxes

**Fig 1:**
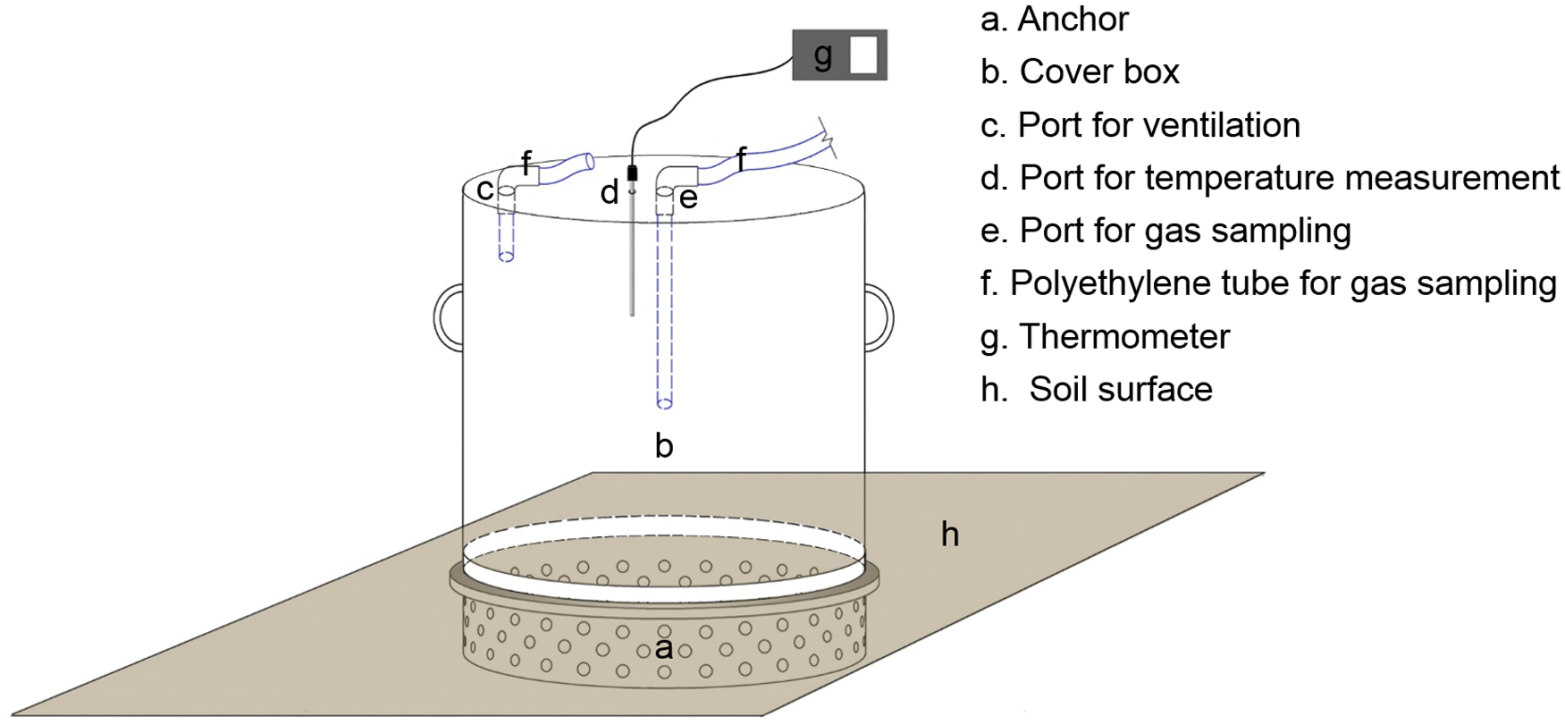
Design of the static chamber used for measuring N_2_O and CH_4_ fluxes.

A static chamber was used to simultaneously measure CH_4_ and N_2_O fluxes as described by Zheng et al. [21]. The chamber was comprised of two parts: a cylindrical steel anchor and a cover (Fig 1). The heights of the anchor for rice and vegetables were 18 and 13 cm, respectively, and internal diameter was 50 cm. The cover had heights of 40 cm (used for vegetables) and 70 cm (used for rice) and internal diameter was 50 cm. The anchor was inserted into the soil to a depth of 15 cm for rice and 10 cm for vegetables (Fig 1). The bottom part of the anchor inserted into the soil contained holes for water and nutrient exchange between inside and outside of the anchor. The groove on the upper part of the anchor was equipped with a sealing strip to ensure that gas does not leak from the joint of the anchor and the cover box when gas samples were collected. The anchor was kept in place throughout the entire study period, except during tillage and planting. Five seedlings of rice or three seedlings of vegetables were planted inside the anchor. The planting density inside the chamber was similar to that of the crop planted outside. The cover had ports for ventilation and gas sampling (Fig 1). The cover was placed tightly above the anchor during gas sampling and removed after sampling. Before starting the experiment, measurements of CH_4_ and N_2_O fluxes in the bare soil from the chambers with two different heights showed that the height had no significant effect on gas fluxes. A tin foil reflective coating was used to cover the chamber to minimize solar heating and fluctuations in the headspace temperature.

Five gas samples were collected from each chamber using 100 ml plastic syringes at 10-min interval twice each week between 8:00 AM and 12:00 PM throughout the growing season. Immediately after tillage, fertilization, irrigation or drainage, however, samples were collected daily for one week. The gas samples were analyzed using a gas chromatograph (GC System, 7890A, Agilent Technologies) equipped with an electron capture detector (ECD) and a flame ionization detector (FID). The CH_4_ and N_2_O fluxes were calculated from linear or nonlinear changes in the gas concentrations over time [22, 23]. The lowest accepted correlation coefficient (r) value for the regression of CH_4_ and N_2_O evolutions was 0.87. When the absolute value of r from the nonlinear model minus that from the linear model was equal to or less than 0.0002, the fluxes from the linear model were adopted and vice versa.

### Measurement of environmental factors and yields

The air temperature inside the chamber headspace was measured with a thermometer placed inside the chamber when the gas samples were collected. The soil temperature and water content were recorded to a 5 cm depth with a probe installed near the chamber, and the data were collected with an automatic data logger. The standing water depth in the rice fields was monitored using a steel ruler when the fields were flooded. The air temperature and precipitation data were collected from an on-site automatic meteorological station adjacent to the experimental plots. Both vegetable and rice were manually harvested. All of aboveground crop biomass was harvested and no harvested crop straw was incorporated into the soil. To determine yields, the weights of the rice grain and vegetables were recorded from a 1 m^2^ area at harvest. The grain or vegetable samples, which had masses of approximately 100 g, were oven dried to a constant weight at 65°C for 24 h after determining the wet weight, which was used as conversion factor to determine rice grain and vegetable yields.

### Data analysis

The seasonal or annual cumulative emissions of CH_4_ and N_2_O were computed as the sum of the daily CH_4_ and N_2_O fluxes, respectively. For the days when measurements were not obtained, we calculated the emissions by linear interpolation using the data from nearest days. The total GWP (g CO_2 eq_ m^−2^) impact was calculated as = (CH_4_ emissions × 25) + (N_2_O emissions × 298). A direct emission factor (EF_d_) of applied N fertilizer was calculated for N_2_O as the difference between the total emissions from the fertilized and the unfertilized treatments divided by the amount of N applied [24–26].

A one-way analysis of variance (ANOVA) with a Duncan test to separate means was used to analyze the difference in the soil properties between the treatments prior to the conversion. An analysis of the repeated measures in the mixed model was used to analyze the data on the CH_4_ and N_2_O fluxes, where land use and fertilization were set as fixed effects, replication as a random effect and the sampling time as a repeated measure variable. The model was also used to analyze the differences in the crop yields and GWPs among the treatments. Means were separated by using Duncan’s test when the treatments and interactions were significant. Exponential or linear regression analyses were used to identify correlations between the gas fluxes and the soil temperature or water content. The differences were considered statistically significant at *P* < 0.05.

## Results and Discussion

### Environmental variables and crop yields

**Fig 2:**
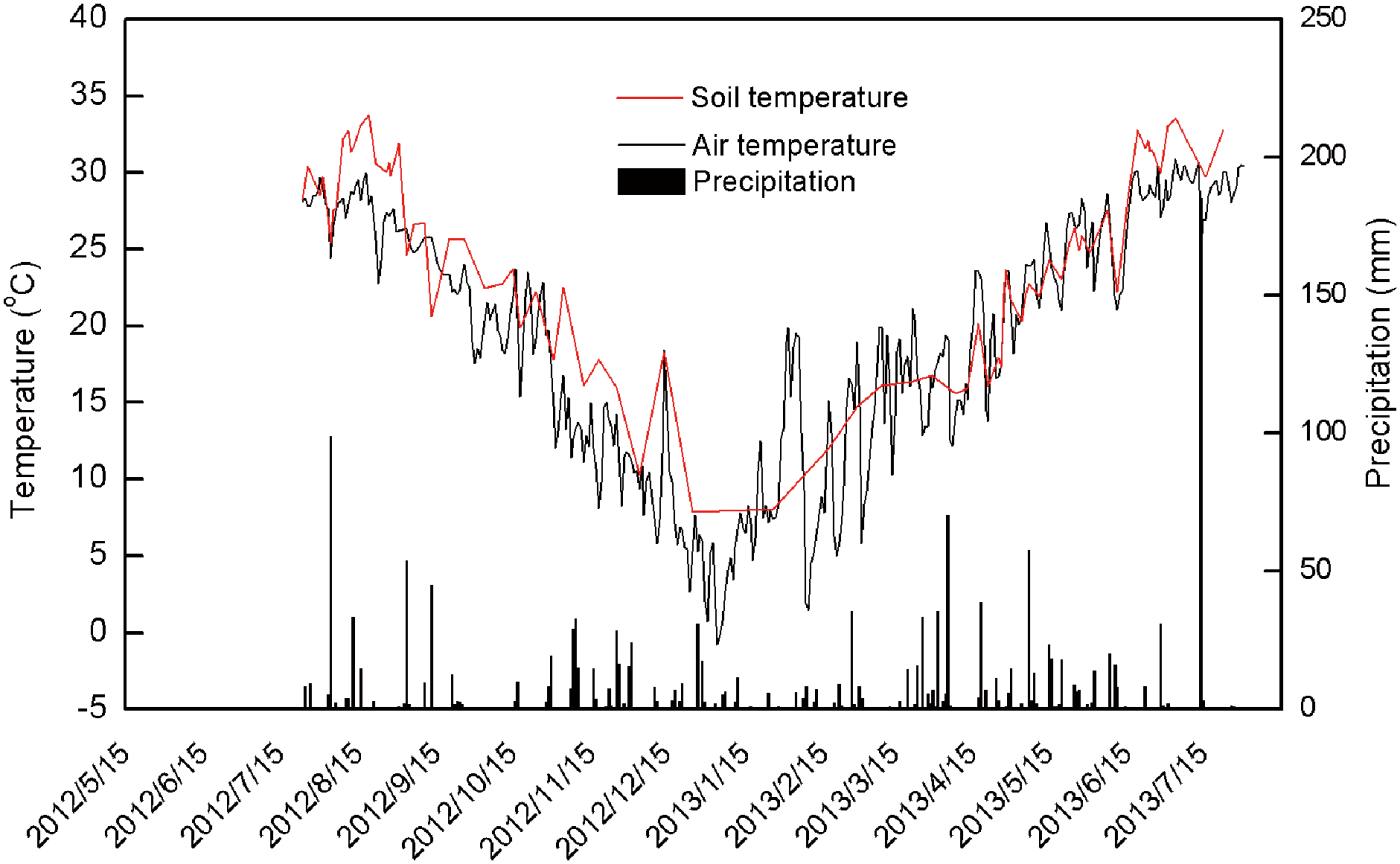
Daily air temperature, soil temperature at the 0–5 cm depth, and precipitation from May 2012 to July 2013 at the study site.

Air and soil temperatures showed similar seasonal variations during the experimental period (Fig 2). Maximum (approximately 31°C) and minimum (approximately −1°C) air temperatures were observed in July and January, respectively. Similarly, soil temperature reached maximum (approximately 34°C) and minimum (approximately 8°C) values in August and December, respectively. Lag effects of soil temperature had been observed in previous studies [27]. Total annual precipitation was 1537 mm. The number of precipitation days was 177, with intense precipitation during August-September 2012 and April-May 2013.

The rice and vegetables yields are presented in Table 3. The land use had a significant effect on crop yields. Yield was greater with rice than vegetables in seasons 1 and 3. Fertilization and its interaction with land use had no effect on the crop yields. Increased soil residual N, P, and K after the harvest of the previous crop may have resulted in non-significant effect of fertilization on crop yields [28], as fertilization rates to crops in each year were not adjusted to residual nutrient levels.

**Table 3:** Rice and vegetables yields as affected by treatment in each growing season. The different superscript letters within the same column between different treatments indicate significant differences.

Treatment^†^	Yields (kg ha^-1^)			
	Season 1 (Late rice vs. cowpea)	Season 2 (Fallow vs. white radish)	Season 3 (Early rice vs. pepper)	Annual yields
RF	4076±191 ^a^^‡^	0 ^b^	4510±605 ^a^	8586±465 ^a^
RNF	4760±378 ^a^	0 ^b^	3081±446 ^a^	7842±799 ^a^
VF	1007±78 ^b^	4966±947 ^a^	95±26 ^b^	6069±958 ^b^
VNF	919±125 ^b^	3028±125 ^a^	99±37 ^b^	4046±229 ^b^
**Land use**	*P* <0.01	*P* <0.01	*P* <0.01	*P* <0.01
**Fertilization**	*P* = 0.20	*P* = 0.07	*P* = 0.08	*P* = 0.06
**Land use × Fertilization**	*P* = 0.11	*P* = 0.07	*P* = 0.08	*P* = 0.36

^†^ Treatments are RF, rice with fertilization; NRF, rice with no fertilization; VF, vegetables with fertilization, and VNF, vegetables with no fertilization.

^‡^ Numbers followed by different letters (a, b) within a column are significantly different at *P* = 0.05 by the leasy significant difference test.

### Methane flux

**Fig 3:**
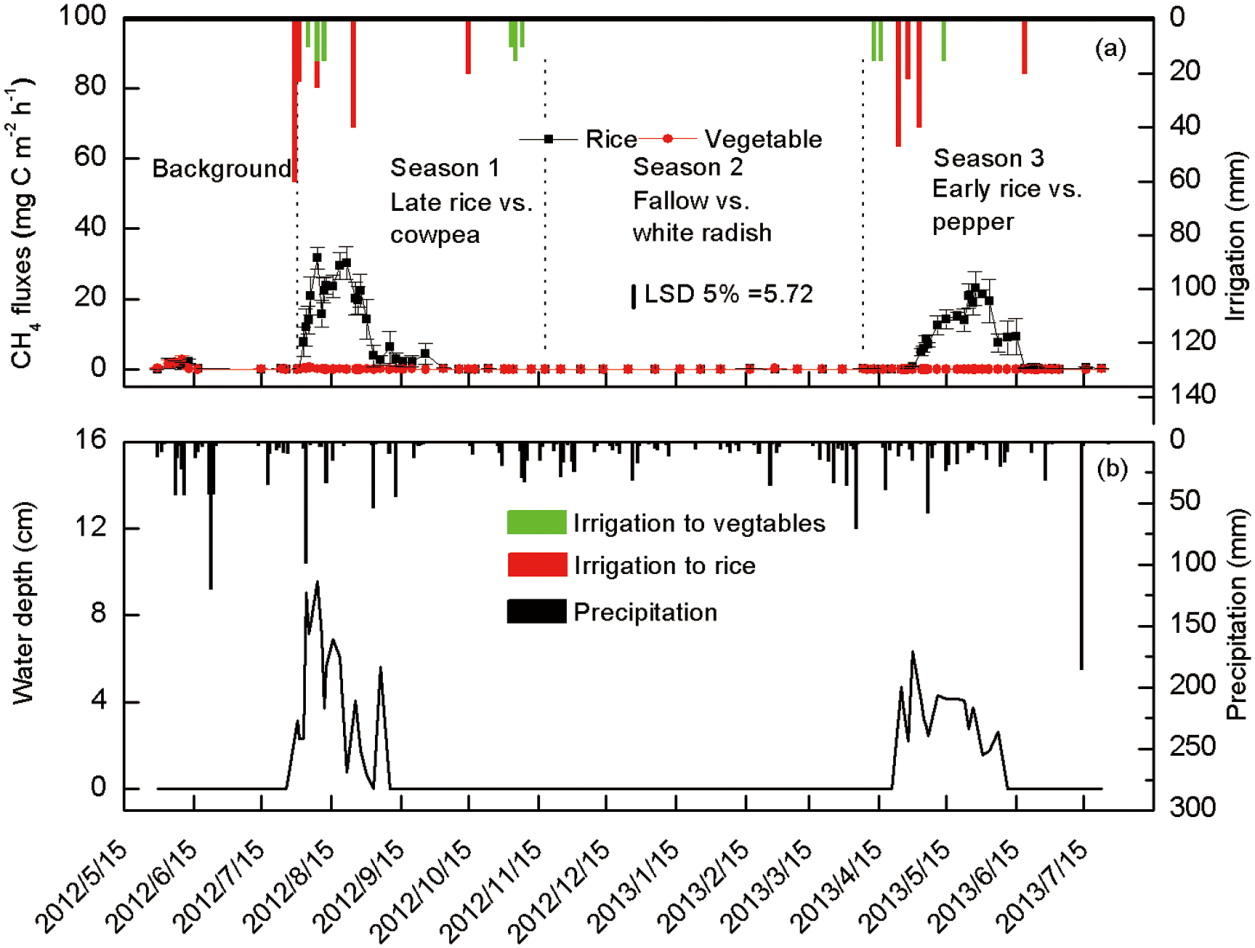
Seasonal variations in the CH_4_ fluxes and the amount of irrigation for rice and vegetables (a) and the water depth under rice and daily precipitation from May 2012 to July 2013 (b).

The land-use, date of gas sampling, and their interaction significantly affected CH_4_ fluxes (Table 4). Under vegetables, CH_4_ flux remained at very low levels throughout the year, ranging from −0.09 to 0.49 mg C m^−2^ h^−1^ (Fig 3). The CH_4_ flux under late rice peaked during August 2012 and under early rice during May 2013 when standing water level was high. The maximum flux was also greater with late rice (31.6 mg C m^−2^ h^−1^) than early rice (23.0 mg C m^−2^ h^−1^). During November 2012 to March 2013 when no rice was grown and standing water was absent, CH_4_ flux dropped to negative values.

**Table 4:** Results from the linear mixed model on the effects of land use and fertilization on the CH_4_ and N_2_O fluxes.

Source of variation	CH_4_		N_2_O	
	*F*-values	*P*-values	*F*-values	*P*-values
Sampling date (S)	7.61	<0.01	51.98	<0.01
Land use (L)	33.69	<0.01	249.95	<0.01
Fertilization (F)	2.60	0.11	145.35	<0.01
L × F	0.67	0.41	86.74	<0.01
S × L	13.89	<0.01	13.95	<0.01
S × F	0.06	0.94	19.36	<0.01
S × L × F	1.18	0.31	16.36	<0.01

Greater standing water level and soil temperature increased CH_4_ flux under rice than vegetables and under late than early rice. Anaerobic decomposition of soil organic matter in standing water increased CH_4_ flux under rice compared with vegetables where no standing water was present. Greater CH_4_ flux in late than early rice was related to higher standing water level due to intense precipitation and increased soil temperature in August 2012 than May 2013 (Fig 2). Previous studies have found that CH_4_ flux showed a positive response to increased temperature due to enhanced microbial activity [29]. Berger et al. [30] found that standing water level has direct influence on CH_4_ emissions. Fertilization with N, P, and K had little influence on CH_4_ flux. Dan et al. [31] found that N fertilization stimulated both CH_4_ production and CH_4_ oxidation, resulting in no significant effect on CH_4_ emissions.

### Nitrous oxide flux

**Fig 4:**
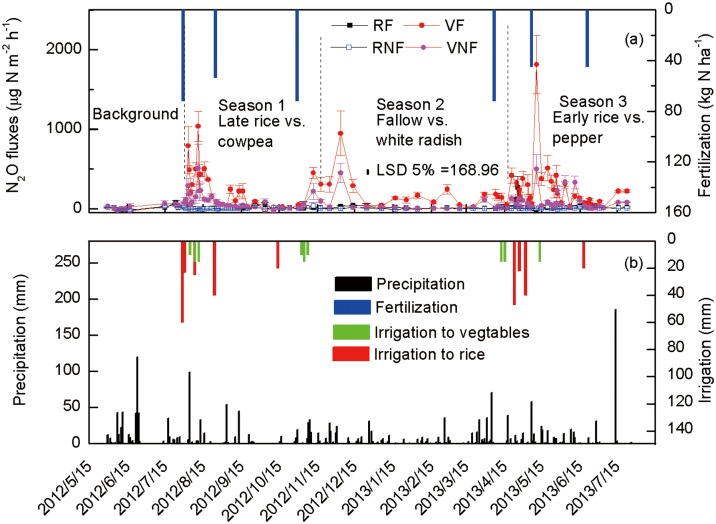
Seasonal variations in the N_2_O fluxes under rice and vegetables with or without chemical fertilization, the fertilization time, irrigation water, and daily precipitation from May 2012 to July 2013. Treatments are RF, rice with fertilization; NRF, rice with no fertilization; VF, vegetables with fertilization, and VNF, vegetables with no fertilization.

**Fig 5:**
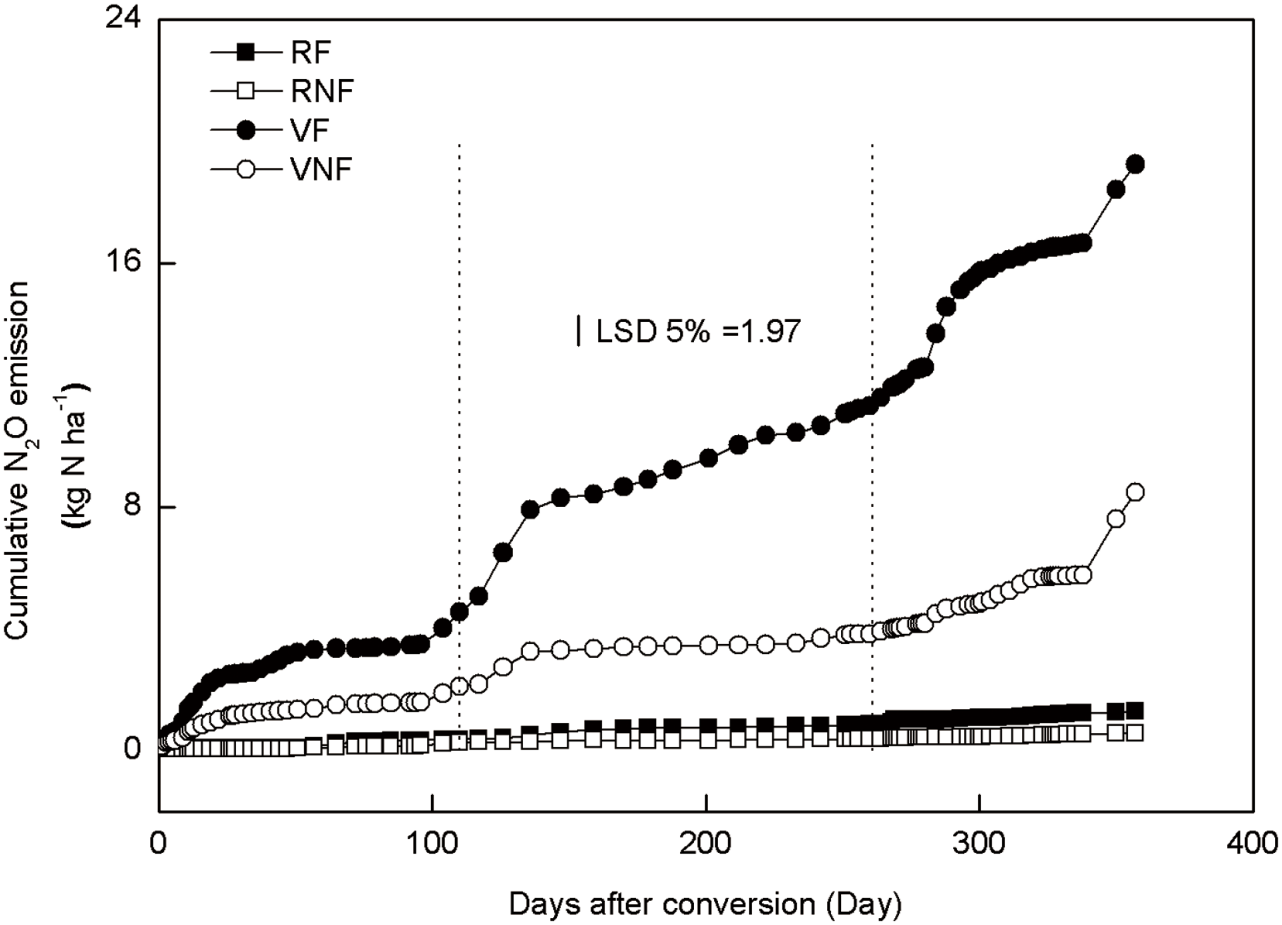
Cumulative N_2_O emissions under rice and vegetables with or without fertilization during the study period. Treatments are RF, rice with fertilization; NRF, rice with no fertilization; VF, vegetables with fertilization, and VNF, vegetables with no fertilization.

Land-use, fertilization, date of gas sampling and their interactions were significant for N_2_O flux (Table 4). The vegetable fields had significantly higher N_2_O flux than the rice fields. The N_2_O fluxes under vegetables primarily peaked after N fertilization and/or irrigation and precipitation (Fig 4). Nitrogen fertilization increased cumulative N_2_O emissions for the vegetable fields by 126% but not for the rice fields (Fig 5). Maximum N_2_O peak of 1811 μg N m^-2^ h^-1^ was observed under pepper in May 2013 compared with 1036 μg N m^-2^ h^-1^ under cowpea in August 2012 and 947 μg N m^-2^ h^-1^ under white radish in December 2012. Immediately after heavy precipitation and/or irrigation in August and November 2012 and May 2013, N_2_O fluxes peaked under unfertilized vegetables similar to those observed for fertilized vegetables.

Reduction of N_2_O to N_2_ through the denitrification process in the anaerobic environment may have decreased N_2_O flux under rice compared with vegetables [8]. Another possible reason is that increased N uptake by rice due to higher yield (Table 3) may have reduced soil NO_3_-N and therefore resulted in lower N_2_O flux under rice than under vegetables. The increased N_2_O flux immediately after planting under unfertilized vegetables could be the results of soil disturbance due to tillage [32], followed by increased soil water availability due precipitation and irrigation. Many previous studies observed N_2_O peak events after precipitation [33, 34]. Enhanced microbial activity due to increased soil water content as a result of precipitation and irrigation can increase soil organic matter mineralization and therefore accelerate N_2_O emissions [35].

### Aggregate emissions of methane and nitrous oxide

**Fig 6:**
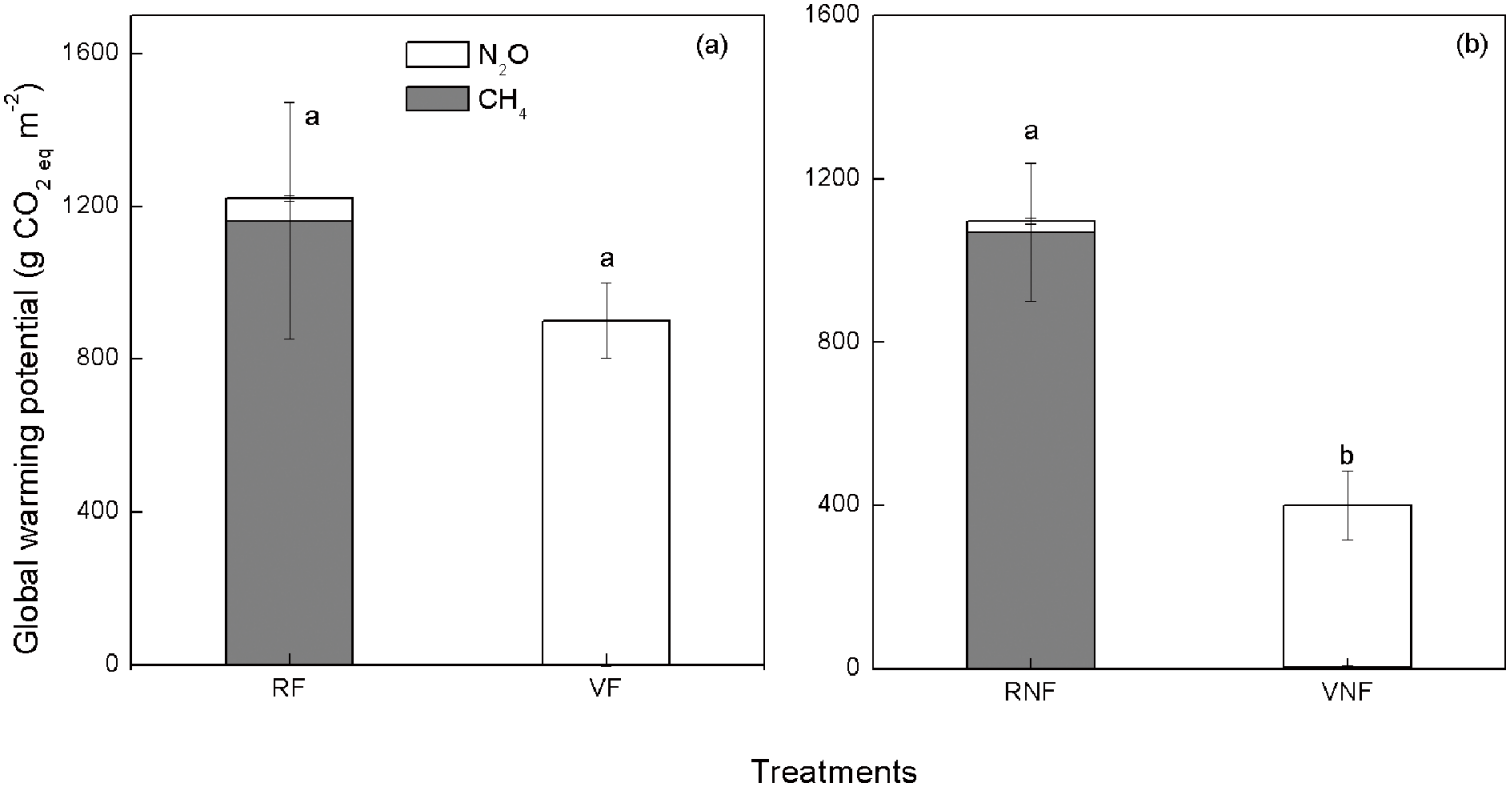
Global warming potentials under rice and vegetables with or without chemical fertilization. Treatments are RF, rice with fertilization; NRF, rice with no fertilization; VF, vegetables with fertilization, and VNF, vegetables with no fertilization. Bars with different letter at the top are significantly different at *P* = 0.05 by the least significant difference test.

The GWP was lower under vegetables than under rice when unfertilized, but not different between land uses when fertilized, (Fig 6a and 6b). This is because rice produced higher CH_4_ flux than vegetables, regardless of fertilization (Fig 3). In contrast, N_2_O flux was higher under vegetables than under rice and higher with fertilized than unfertilized vegetables (Figs 4 and 5). With or without fertilization, GWP did not alter under rice, but GWP was greater with fertilization than without under vegetables. This suggests that rice and vegetables can be produced by applying chemical fertilizers without emitting net greenhouse gases.

Different management approaches should be applied for rice and vegetable fields to reduce the GWP. Field should be regularly drained in rice to control CH_4_ emissions and N fertilization rate should be reduced in vegetables to reduce N_2_O emissions while maintaining yields. Many studies have shown that water management is one of the most effective options for decreasing CH_4_ emissions from rice fields [36, 37]. Short-term draining can be an effective way to significantly reduce CH_4_ emissions [38]. Nitrogen fertilization has already been considered as the best predictor for N_2_O emissions [39]. Nitrogen fertilization rates of 550 to 600 kg N ha^−1^ yr^−1^ have been used for intensive cropping systems in China which do not increase crop yields but degrade soil and environmental quality by increasing soil acidification, N_2_O emissions, and N leaching to the groundwater [40]. In our study, N fertilization increased N_2_O emissions, but had no significant effect on the crop yields. One approach to reduce N rate is to apply N fertilizer based on soil NO_3_-N test to a depth of 60 cm and N mineralization potential. Other is to grow legume cover crops that supply N to the soil.

## Conclusions

Land-use conversion from double rice cultivation to vegetables mitigated CH_4_ emissions but increased N_2_O fluxes. Fertilization with N, P, and K increased N_2_O fluxes under vegetables, but not under rice. In contrast, fertilization had no effect on CH_4_ fluxes, regardless of land use practices. The GWP was lower under vegetables than rice when not fertilized, but not different between land uses when fertilized. Management practices, such as frequent land draining under rice to reduce CH_4_ emissions and reducing N fertilization rate by applying N fertilizer based on soil test and growing legume cover crop to reduce N_2_O emissions can reduce GWP under rice and vegetables while maintaining yields.

## Supporting Information

S1 Dataset Data underlying the findings described in the manuscript. (XLSX)
